# A Regression Model to Predict Linezolid Induced Thrombocytopenia in Neonatal Sepsis Patients: A Ten-Year Retrospective Cohort Study

**DOI:** 10.3389/fphar.2022.710099

**Published:** 2022-02-03

**Authors:** Lufen Duan, Qin Zhou, Zongtai Feng, Chenqi Zhu, Yan Cai, Sannan Wang, Meiying Zhu, Jingjing Li, Yunlong Yuan, Xin Liu, Jiantong Sun, Zuming Yang, Lian Tang

**Affiliations:** ^1^ Department of Pharmacy, The Affiliated Suzhou Hospital of Nanjing Medical University, Suzhou Municipal Hospital, Suzhou, China; ^2^ Neonatology Department, The Affiliated Suzhou Hospital of Nanjing Medical University, Suzhou Municipal Hospital, Suzhou, China; ^3^ Medical Laboratory, The Affiliated Suzhou Hospital of Nanjing Medical University, Suzhou Municipal Hospital, Suzhou, China

**Keywords:** linezolid-induced thrombocytopenia, neonatal sepsis patients, regression model, linezolid, trough concentration

## Abstract

**Background:** Linezolid-induced thrombocytopenia (LIT) is the main factor limiting the clinical application of linezolid (LZD). The incidence and risk factors of LIT in neonatal patients were possibly different from other populations based on pathophysiological characteristics. The purpose of this study was to establish a regression model for predicting LIT in neonatal sepsis patients.

**Methods:** We retrospectively included 518 patients and divided them into the LIT group and the non-LIT group. A logistic regression analysis was used to analyze the factors related to LIT, and a regression model was established. A receiver operating characteristic (ROC) curve was drawn to evaluate the model’s predictive value. We prospectively collected 39 patients’ data to validate the model and evaluate the effect of LZD pharmacokinetics on LIT.

**Results:** Among the 518 patients, 103 patients (19.9%) developed LIT. The Kaplan–Meier plot revealed that the overall median time from the initiation of LZD treatment to the onset of LIT in preterm infants was much shorter when compared with term infants [10 (6, 12) *vs*. 13 (9.75, 16.5), *p* = 0.004]. Multiple logistic regression analysis indicated that the independent risk factors of LIT were lower weight at medication, younger gestational ages, late-onset sepsis, necrotizing enterocolitis, mechanical ventilation, longer durations of LZD treatment, and lower baseline of platelet level. We established the above seven-variable prediction regression model and calculated the predictive probability. The ROC curve showed that the predicted probability of combined body weight, gestational age, duration of LZD treatment, and baseline of platelet had better sensitivity (84.4%), specificity (74.2%), and maximum AUC (AUC = 0.873). LIT occurred in 9 out of 39 patients (23.1%), and the accuracies of positive and negative predictions of LIT were 88.9 and 76.7%, respectively. Compared with the non-LIT patients, the LIT patients had higher trough concentration [11.49 (6.86, 15.13) *vs.* 5.51 (2.80, 11.61) mg/L; *p* = 0.028] but lower apparent volume of distribution (Vd) [0.778 (0.687, 1.421) *vs.* 1.322 (1.099, 1.610) L; *p* = 0.010].

**Conclusion:** The incidence of LIT was high in neonatal sepsis patients, especially in preterm infants. LIT occurred earlier in preterm infants than in term infants. The regression model of seven variables had a high predictive value for predicting LIT. LIT was correlated with higher trough concentration and lower Vd.

## Introduction

Neonatal sepsis is one of the main causes of morbidity and mortality in preterm infants. Gram-positive pathogens infection remains one of the most common and troublesome causes of nosocomial infections in Neonatal Intensive Care Units (NICUs) (Kocher et al., 2010). Vancomycin is classically known as the first-choice antibiotic for the treatment of Gram-positive pathogens (Rybak et al., 2020). However, the application of vancomycin in neonatal patients has some disadvantages: 1) emerging pathogens with vancomycin resistance (glycopeptide-resistant *Enterococcus faecium* and CoNS or *Staphylococcus aureus* with reduced vancomycin susceptibility); and 2) uncertainty about side effects, in particular, nephrotoxicity and ototoxicity, especially pertinent for preterm infants (Kocher et al., 2010; Sicard et al., 2015; Thibault et al., 2019; Wang et al., 2019; Marissen et al., 2020). LZD remains highly sensitive to drug-resistant Gram-positive organisms, including vancomycin-resistant *Enterococcus faecium*, multi-drug-resistant *Streptococcus pneumoniae*, and methicillin-resistant *Staphylococcus aureus* (Beibei et al., 2010; Kocher et al., 2010; Thibault et al., 2019). Moreover, linezolid had less incidence of ototoxicity and nephrotoxicity when compared with vancomycin, and there is no need to adjust the dose of linezolid in patients with renal insufficiency (Beibei et al., 2010). The available literature suggests that LZD is an effective and a better alternative to vancomycin for the treatment of multidrug-resistant Gram-positive bacterial infections in neonates (Beibei et al., 2010; Kocher et al., 2010; Thibault et al., 2019). Our previous research evaluated the clinical efficacy and safety of vancomycin compared with LZD for the treatment of neonatal Gram-positive bacterial sepsis. The results showed that the clinical effect of vancomycin and LZD was comparable (Tang et al., 2016a).

However, one of the major side effects of LZD is myelosuppression, especially for thrombocytopenia, which is common in adults. The reported rates of linezolid-induced thrombocytopenia (LIT) in adults range from 16.8 to 42.9%, especially higher in impaired renal function patients (Tang et al., 2016a; Crass et al., 2019; Giunio-Zorkin and Brown, 2019; Rao et al., 2020). LIT is the leading cause of LZD withdrawal, prolonged hospital stay, increased blood product infusion, and significantly increased bleeding events and mortality (Giunio-Zorkin and Brown, 2019). Some literature reported that creatinine clearance, the duration of LZD therapy, and trough concentration were the risk factors for LIT in the adult population (Tang et al., 2016a; Crass et al., 2019; Giunio-Zorkin and Brown, 2019; Rao et al., 2020). Therapeutic Drug Monitoring (TDM) can reduce the incidence of LIT and improve LZD dosing regimens and safety in current clinical practice, and the target trough concentration of LZD is recommended as 2–7 μg/ml (Tang et al., 2016a; Kim et al., 2019).

Based on the maturational differences of organs in neonates, there is large inter- and intra-individual variability in pharmacokinetics (Pauwels and Allegaert, 2016). Therefore, the safety data on linezolid in adults are not necessarily suitable for neonates. However, the PK/PD study of LZD and safety studies are limited in this population (Thibault et al., 2019). LIT was mostly reported as case reports in the neonatal population (Abdul-Aziz et al., 2020). Only a study of small sample size (*n* = 32) showed that platelets decreased significantly after the administration of LZD when compared with those before the administration (*p* = 0.03) (Li and Lu, 2011). There have been no reports on the incidence, clinical characteristics, or high-risk factors for LIT in neonates. The results of our previous investigation study (Tang et al., 2016b) indicated that 27 out of 195 (13.8%) neonates were assessed as LIT. The total bilirubin was higher and the platelet count was lower after LZD treatment when compared with vancomycin, with significant differences (*p* = 0.034, *p* = 0.000) (Narayanan et al., 2019). The logistics regression analysis showed that the gestational ages, baseline of platelet (PLT), albumin, and total bilirubin were independent risk factors of LIT (Tang et al., 2016a; Tang et al., 2016b). It is necessary to analyze how to improve the safety of LZD treatment in neonatal patients. The purposes of our study were to establish a regression model for the prediction of LIT in neonatal sepsis patients and evaluate the correlation of LZD trough concentration and pharmacokinetics with LIT.

## Materials and Methods

This was a ten-year retrospective cohort study performed in critically ill neonates hospitalized in the Neonatal Intensive Care Unit (NICU) from June 2009 to December 2019 at the Affiliated Suzhou Hospital of Nanjing Medical University. This hospital is a teaching hospital of Nanjing Medical University, located in Suzhou, Jiangsu province, China, and contains the Suzhou Maternal and Child Care Health Centre. The neonatal unit has a total capacity of 111 beds, including 41 beds in the NICU. This study was approved by the research ethics committee of this hospital (L20129901).

### Study Design, Population, and Data Collection

A neonate aged 0–28 days who met the selection criteria was enrolled in the study. Inclusion criteria were as follows: 1) patients were diagnosed with neonatal sepsis and the presence of neonatal sepsis was determined by positive blood culture and classified as early onset ( ≤72 h) or late onset ( >72 h) (Bozorgmehr et al., 2018). 2) The blood culture pathogens were methicillin-resistant positive bacteria and treated with LZD. We included the first episode of coagulase-negative *Staphylococcus* (CoNS) bacteraemia in each neonate who met the following criteria: two positive blood cultures within 4 days, three within 7 days, or four within ten days. All positive CoNS cultures obtained within 21 days of each other were considered single infectious episodes (Jean-Baptiste et al., 2011). Exclusion criteria were as follows: 1) patients with incomplete or missing clinical and laboratory information; for example, platelet count was not recorded before or after LZD therapy; 2) the duration of LZD treatment was less than 7 days; 3) the baseline of platelet was less than 100 × 10^9^/L; 4) there is disseminated intravascular coagulation (DIC) or other hematological disease or organ bleeding before LZD treatment; and 5) patients are discharged to other hospitals during LZD treatment, as the safety could not be evaluated. Patient data were obtained from the Medical Database in March 2020. Clinical diagnoses were based on the International Classification of Diseases 11th Revision (ICD-11) (World Health Organization, 2019). Neonatal complications were identified by ICD-11 codes (World Health Organization, 2019) and included: asphyxia (ICD-11 code: KB21), respiratory failure (ICD-11 code: KB2D), respiratory distress syndrome (RDS) (ICD-11 codes: KB23.0Z, KB23.0Y, KB23.00, KB23.01, and KB23.02), neonatal pneumonia (ICD-11 code: CA40.Z), sepsis (early- and late-onset sepsis were defined by positive blood cultures before or after 72 h of age) (ICD-11 code: KA60), sepsis shock (ICD-11 code: 1G41), modified Bell’s stage ≥ⅡA necrotizing enterocolitis (NEC) (ICD-11 codes: KB88.1, KB88.2, and KB88.3), purulent meningitis, patent ductus arteriosus (PDA) (ICD-11 code: LA8B.4), bronchopulmonary dysplasia (BPD) (ICD-11 code: KB29.0) defined as a requirement for supplemental oxygen at 36 weeks of postmenstrual age (PMA), intracranial hemorrhage (ICH) (ICD-11 code: KA82.Z), gastrointestinal hemorrhage (ICD-11 code: KA83.1), pneumorrhagia (ICD-11 code: KB28.Z), multiple organ dysfunction syndrome (MODS) (ICD-11 code: MH16), heart failure (ICD-11 codes: KB40.Y and KB40.Z), pulmonary arterial hypertension (ICD-11 code: BB01.0), ileus (ICD-11 code: KB87.3), retinopathy of prematurity (ROP) (ICD-11 code: 9B71.3), and preterm infants (born at <37 weeks of gestation) (ICD-11 code: KA21.4Z). Each outcome was coded as a binary (1/0) variable.

On admission, demographic data [postnatal age (PNA), sex, weight, and gestational age (GA)], infectious diseases, and complications (asphyxia, respiratory failure, RDS, neonatal pneumonia, sepsis, sepsis shock, NEC, purulent meningitis, PDA, BPD, ICH, gastrointestinal hemorrhage, pneumorrhagia, MODS, heart failure, pulmonary arterial hypertension, ileus, and ROP) were registered. Apgar score, blood glucose, white blood cell, hemoglobin, platelet, albumin, alanine aminotransferase, total bilirubin, creatinine, mechanical ventilation, dosage and duration of LZD treatment, the use of other antibacterial drugs, treatment and recovery after thrombocytopenia, and other adverse reactions of LZD (hyperlactic acidemia, gastrointestinal reactions, 5-HT_3_ syndrome, *etc*.) were recorded.

### Dosage Regimen of LZD

The empirical standard dosage regimens of LZD injection (Zyvox; Pfizer Inc., New York, NY, United States) in neonatal sepsis patients were given based on LZD manufacturer’s instructions: 1) preterm neonates with a GA < 34 weeks and a PNA < 7 days, 10 mg/kg, q12h; 2) term infants, preterm neonates with a GA ≥ 34 weeks, and preterm neonates with a GA < 34 weeks and a PNA ≥ 7 days, 10 mg/kg, q8h. The therapy duration was 10–14 days. Considering clinical efficacy, adverse reactions, and other factors, the duration may be ended early or extended appropriately.

### Definitions of Thrombocytopenia, Anemia, Neutropenia, and Groups

The definition of thrombocytopenia is a platelet count of < 150 × 10^9^/L. It can be classified as mild thrombocytopenia (platelet count at 100–150 × 10^9^/L), moderate thrombocytopenia (platelet count at 50–100 × 10^9^/L), and severe thrombocytopenia (platelet count < 50 × 10^9^/L) (Gunnink et al., 2014; Cremer et al., 2016). Patients with hematological diseases or baseline of PLT < 150 × 10^9^/L were excluded from this analysis. Only DIC and bleeding events that occurred after LZD treatment were included in this study. The changes in the hematological parameters were assessed every 2–4 days during LZD therapy. The definition of LIT is a drop in platelet count below 75% of the baseline level or below 150 × 10^9^/L after the administration of LZD for more than seven days. The definition of anemia is a decrease in hemoglobin concentration ≥ 2 g/dL from the baseline, and the definition of neutropenia is absolute neutrophil count (ANC) < 0.5 × 10^9^/L (Nukui et al., 2013). LIT would not be diagnosed if PLT levels decreased by more than 25% during the LZD treatment and increased again without medication that elevated platelets. According to the definition of LIT, patients after LZD treatment were divided into the LIT group and the non-LIT group.

### Establishment of the Regression Model

Univariate and multivariate logistic regression analyses were used to select the covariables of the regression model. In the univariate analysis, the χ^2^ test or Fisher’s exact test was used to compare the categorical variables. The continuous variables were compared using the Mann–Whitney *U* test. The covariates with *p* value < 0.1 were included in the multivariate logistic regression analysis (backward procedure, based on *p* value of predictor removed). Statistical Package for the Social Sciences (SPSS) software automatically screened out the independent variables when the optimal balance was reached between the fitting degree of the prediction model.

Independent risk factor variables were used to establish regression equations and calculate predicted probabilities. The receiver operating characteristic (ROC) curves of risk factors and predicted probabilities were drawn. The area under the curve (AUC), cut-off point, Youden’s index, sensitivity, and specificity were used to evaluate their predictive value for predicting LIT.

### Clinical Validation of the Regression Model

We prospectively collected neonatal sepsis patients who received LZD treatment for confirmed Gram-positive infections and measured the steady-state trough concentration from January 2020 to December 2020. Each patient signed the informed consent of this study at the time of enrollment. The prediction probability for each patient was calculated based on the regression model, and the cut-off point of prediction probability was used to determine whether the patients had LIT. The predictive accuracy of this logistic regression model for LIT prediction was expressed as positive prediction accuracy and negative prediction accuracy.

### Determination of LZD Trough Concentration

We measured the steady-state trough concentration (after the fourth maintenance dose and 30 min prior to the next dose). The target serum LZD trough concentrations range from 2 to 7 mg/L, AUC_24h_ at 80–120 mg/Lh, and the safety threshold is AUC_24h_ < 300 mg/Lh (Abdul-Aziz et al., 2020). We took 1 ml of the whole blood, placed it in a yellow tube (including coagulant and separation glue), and sent it to the medical laboratory for centrifugation within 2 h. The supernatant (serum) was separated and stored at −80°C, and the concentration of LZD was determined within three days. The blood concentration of LZD was determined by the liquid chromatography tandem mass spectrometry (LC-MS/MS) method, TRIPLE QUAD 4500MD mass spectrometry (AB SCIEX, United States), Jasper HPLC, and SB-AQ RRHD (50 × 3.0 mm, 1.8 m, Agilent, United States). The Analyst 1.6.1 data processing system is used for analysis. Chromatographic conditions: mobile phase A was 0.1% formic acid aqueous solution. The mobile phase B was 0.1% methanol formate solution; gradient elution; flow rate 0.4 ml/min; injection volume 2 μl; column temperature 45°C; 0–0.5 min, 10% B; 0.5–1.5 min, 10–95% B; 1.5–2.0 min, 95% B; 2.0–2.2 min, 95–10% B; and 2.2–3.0 min, 10%B. Mass spectrometry conditions: electrospray ionization element (ESI) and positive ion detection (MRM). Ion quantitative analysis reaction level: mass-to-charge ratio (M/Z) 338.6 → M/Z 296.2 (LZD), cone voltage 60 V, collision energy 30 eV; (M/Z) 362.2 → (M/Z) 261.1 (internal standard solution, levofloxacin) cone voltage 65 V, and collision energy 35 eV.

### LZD Pharmacokinetic Parameters and Relationship With LIT

We calculated the Vd, clearance rate (CL), and AUC_24h_ of LZD in neonatal sepsis patients using the published population pharmacokinetic (PPK) model of intravenous LZD in preterm infants (Thibault et al., 2019). This PPK model was developed using nonlinear mixed-effects modeling (NONMEM). This model was a one-compartment model, including 78 plasma concentrations collected from 26 infants. This model included PNA and weight on clearance and weight on volume of distribution. The trough concentrations and pharmacokinetic parameters of LZD were compared between LIT and non-LIT groups. The Vd and CL of LZD can be calculated using this published PPK model. The CL, Vd, and AUC_24h_ calculation formula are as follows (Thibault et al., 2019):

$$CL\left(L/h\right)=0.181\times {\left(\frac{WT\left(kg\right)}{1.4}\right)}^{0.405}\times {\left(\frac{PNA\left(days\right)}{0.07}\right)}^{0.831},$$ (1)

$$V_{d}\left(L\right)=1.17\times {\left(\frac{WT\left(kg\right)}{1.4}\right)}^{0.801},$$ (2)

$$AUC_{24h}\left(mg/L\cdot h\right)=\frac{daily\;dose\left(mg\right)}{CL\left(L/h\right)}.$$ (3)

### Statistical Analysis

All statistical analyses were performed using the SPSS, version 22 (SPSS Inc., Chicago, IL, United States), and GraphPad Prism version 9. The categorical variables were summarized as frequencies and proportions (%); Pearson’s chi-square test or Fisher’s exact test was used to analyze categorical data. All the continuous variables were checked for normality using the Shapiro–Wilk test. When these data were not normally distributed, the continuous variables were summarized as the medians and interquartile ranges and Mann–Whitney *U* test was used to analyze these continuous data. When these data were in accordance with normal distribution, the continuous variables were summarized as mean ± SD and a *t*-test was used to analyze these continuous data. Two-tailed *p* values of < 0.05 were considered statistically significant.

## Results

### Patient Characteristics and Univariate Logistic Regression Analysis

**FIGURE 1:**
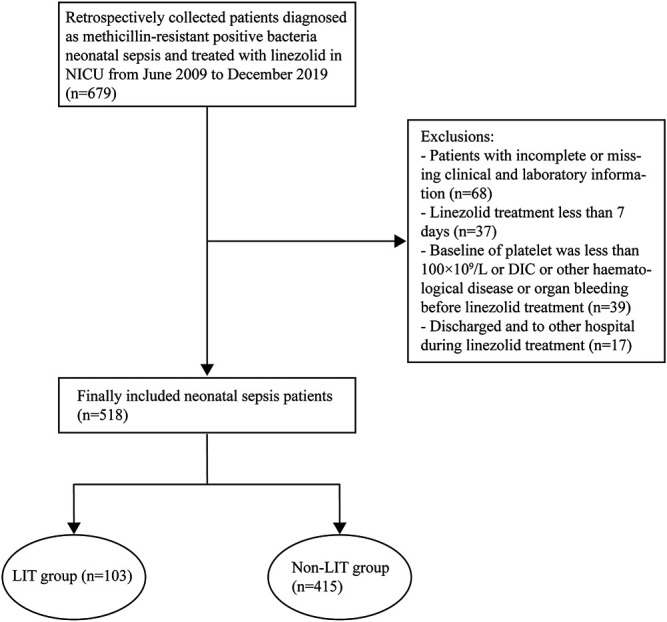
Flowcharts of patients included and excluded from the study. LIT, LZD-induced thrombocytopenia; Non-LIT, non-LZD-induced thrombocytopenia.

**FIGURE 2:**
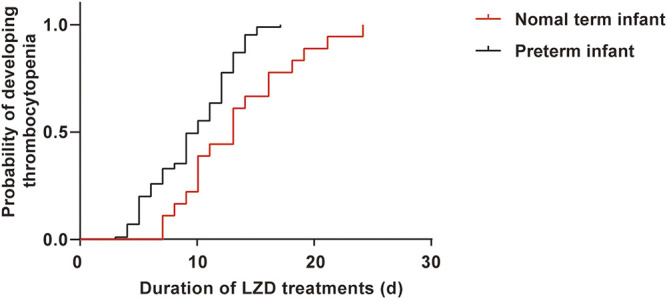
The Kaplan–Meier plot showing the time from initiation of LZD therapy to development of thrombocytopenia in preterm infants and term infants. The time was much shorter in preterm, had significant differences when compared with term infants [10 (6, 12) *vs*. 13 (9.75, 16.5), *p* = 0.004].

A total of 518 neonatal sepsis patients were enrolled, 103 cases developed LIT after LZD treatment, and the incidence of LIT was 19.9% (103/518). The 518 cases were divided into the LIT group (103 cases) and the non-LIT group (415 cases) (Figure 1). The incidence rates of mild, moderate, and severe thrombocytopenia were 32.0% (33/103), 24.3% (25/103), and 43.7% (45/103), respectively. A drop in hemoglobin levels and leukopenia was observed in 37.9% (39/103) and 2.9% (3/103) of LIT patients. Hyperlacticemia was observed in 7.8% (8/103) of LIT patients. There were 85 cases (82.5%) and 254 cases (61.2%) of preterm infants in the LIT patients and the non-LIT patients, respectively, and the difference was statistically significant (*p* < 0.001). The incidence of LIT was much higher in preterm infants than term infants (25.1 *vs*. 10.1%, *p* < 0.001). The Kaplan–Meier plot revealed that the overall median time from the initiation of LZD treatment to the onset of LIT in preterm infants was much shorter when compared with term infants [10 (6, 12) *vs*. 13 (9.75, 16.5), *p* = 0.004] (Figure 2). All 103 cases were discontinued LZD due to LIT. A total of 56 cases received a blood transfusion, 43 cases received human immunoglobulin injection, and 17 cases received PLT transfusion. Platelets returned to normal after LZD withdrawal in 78 cases, and 9 cases with mild thrombocytopenia were not tested for PLT after LZD withdrawal. Platelets did not return to normal in 16 cases. A total of 24 cases had ICH, of which six cases were diagnosed as DIC after LZD treatment in the LIT group, seven cases had gastrointestinal bleeding, and eight cases had a pulmonary hemorrhage.

**FIGURE 3:**
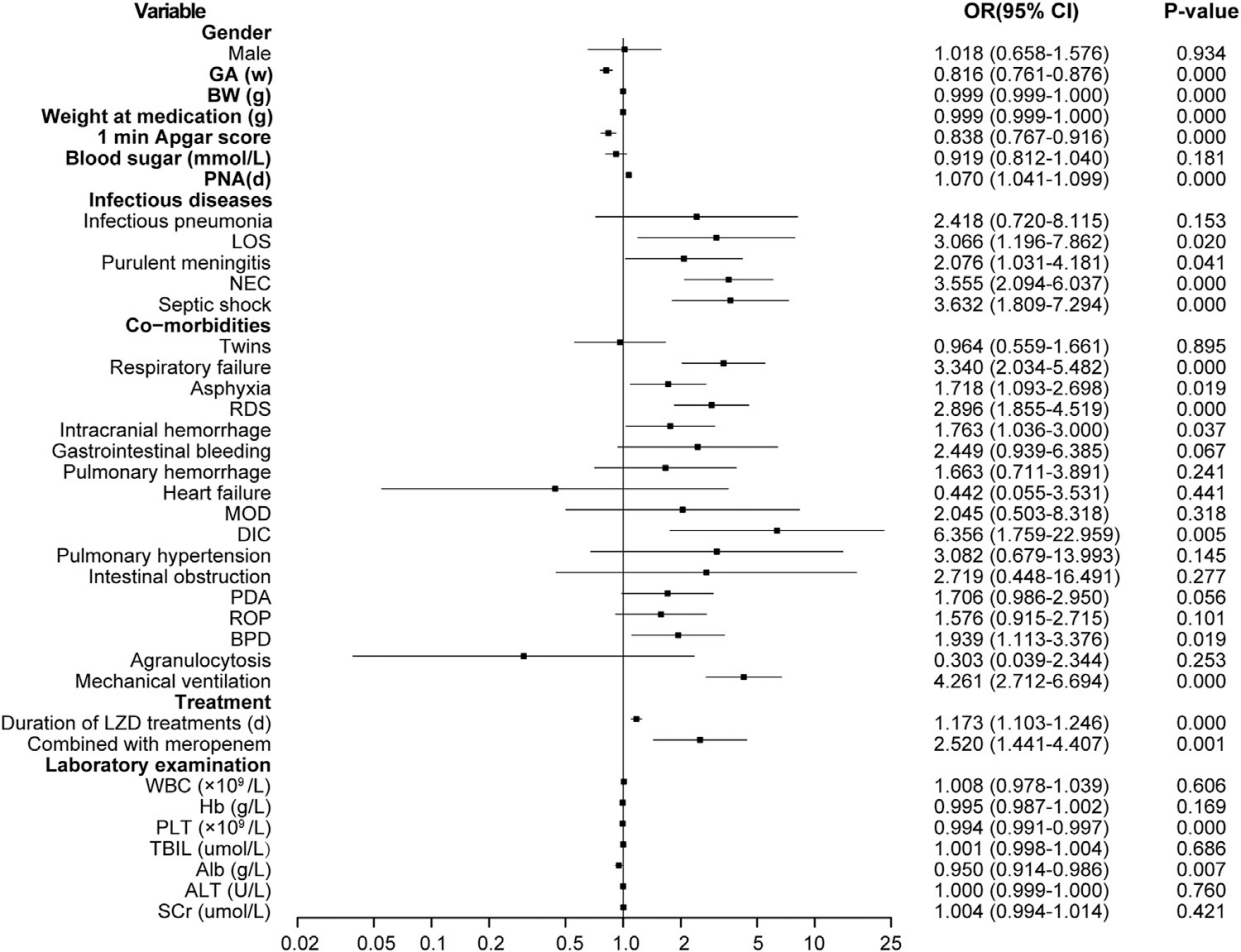
Results of thrombocytopenia risk factors screened by single factor logistic regression analysis. LZD, linezolid; LIT, LZD-induced thrombocytopenia; Non-LIT, non-LZD-induced thrombocytopenia; BW, birth weight; GA, gestational age; PNA, postnatal age; OR, odds ratio; LOS, late-onset sepsis; NEC, neonatal necrotizing enterocolitis; RDS, respiratory distress syndrome; MOD, multi-organ damage; DIC, disseminated intravascular coagulation; PDA, patent ductus arteriosus; ROP, retinopathy of prematurity; BPD, bronchopulmonary dysplasia; WBC, white blood cell; Hb, hemoglobin; PLT, platelet; TBIL, total bilirubin; Alb, albumin; ALT, alanine aminotransferase; SCr, serum creatinine.

According to the univariate analysis, the LIT group had lower birth body weight and weight at LZD medication; younger gestational ages; lower scores of Apgar at 1 min; longer durations of LZD treatment; more underlying conditions, such as late-onset sepsis (LOS), purulent meningitis, NEC, sepsis shock, respiratory failure, asphyxia, RDS, DIC, and ICH after LZD treatment, BPD; more mechanical ventilation; more use of meropenem; and lower baseline of PLT and albumin. There were statistically significant differences when compared with the non-LIT group (*p* < 0.05) (Figure 3).

### Multivariate Logistic Regression Analysis and Logistic Regression Model

**FIGURE 4:**
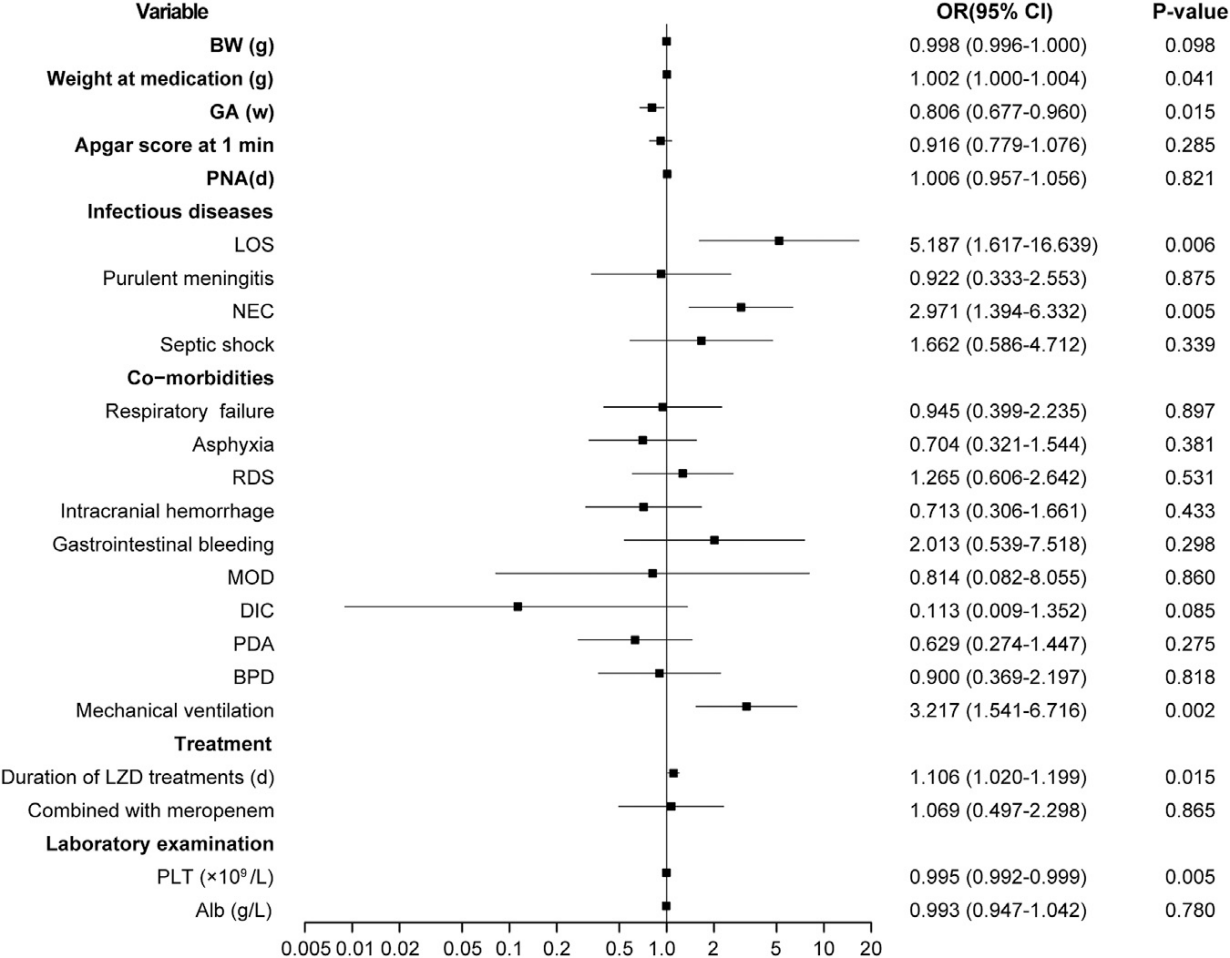
Results of thrombocytopenia risk factors screened by multifactor logistic regression analysis. OR, odds ratio; BW, birth weight; GA, gestational age; PNA, postnatal age; LOS, late-onset sepsis; NEC, neonatal necrotizing enterocolitis; RDS, respiratory distress syndrome; MOD, multi-organ damage; DIC, disseminated intravascular coagulation; PDA, patent ductus arteriosus; BPD, bronchopulmonary dysplasia; LZD, linezolid; PLT, platelet; Alb, albumin.

The risk factors for LIT were estimated by the stepwise multivariate linear regression analysis (backward procedure, based on *p* value of predictor removed) and the identification of the cut-off values with ROC curve analyses. Variables with *p* < 0.1 in the univariate results were estimated in the multivariate analysis. The significant independent factors for the occurrence of LIT were lower weight at LZD medication, younger gestational ages, LOS, NEC, mechanical ventilation, lower baseline of PLT, and longer durations of LZD treatments (*p* < 0.05) (Figure 4). After the introduction and elimination of the above independent risk factors independent variables, the logistic regression equation was finally established:

$$\begin{array}{l} Logit\left(p\right)=0.002\times weight\;at\;linezolid\;medication\\ -0.221\times gestational\;weeks-1.686\times LOS\\ -1.083\times NEC-1.475\times mechanical\;ventilation\\ +0.128\times days\;of\;linezolid\;treatments\\ -0.006\times baseline\;of\;platelet+9.785 \end{array}$$ (4)

### The Models’ Ability to Identify LIT Patients

**FIGURE 5:**
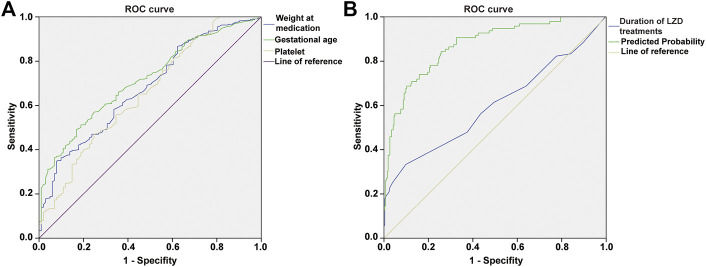
ROC curve of weight at medication, gestational age, platelet **(A)**; duration of LZD treatments, predicted probability **(B)**, independent risk factors and combined predictor for LIT. The predicted probability of combined medication weight (AUC = 0.679), gestational age (AUC = 0.715), platelet (AUC = 0.605), and duration of LZD treatments (AUC = 0.600) had better sensitivity (84.4%) and specificity (74.2%), Youden’s index 0.586, as well as the maximal AUC (AUC = 0.873).

For each independent risk factor of continuous variables, we calculated the specificity and sensitivity of the resulting logistic regression model by constructing ROC curves and calculating the area under the curve (AUC) statistic to estimate the model’s ability to identify LIT patients. The ROC curves of weight at LZD medication, gestational age, PLT, duration of LZD treatment, and predicted probability were drawn. The area under the ROC curve, cut-off point, Youden’s index, sensitivity, specificity of weight at LZD medication, gestational age, PLT, duration of LZD treatments, and predicted probability are shown in Table 1 and Figure 5. The area under the ROC curve of the predicted probability was 0.873, higher than the other four factors, the Youden index was 0.586, and the sensitivity and specificity were 84.4 and 74.2%.

**TABLE 1:** Area under the curve and cut-off values of receiver operating characteristic curve for prediction of thrombocytopenia in neonates on LZD treatment.

Risk factors	AUC (95%confidence interval)	*p*vvalue	Cut-off point	Youden’s index	Sensitivity (%)	Specificity (%)
Weight at medication (g)	0.679 (0.621–0.736)	0.000	2365.0	0.269	34.8	92.1
Gestational age (w)	0.715 (0.662–0.768)	0.000	32.8	0.324	51.2	81.2
PLT (×10^9^/L)	0.605 (0.550–0.660)	0.000	233.5	0.222	47.0	75.2
Duration of LZD treatments (d)	0.600 (0.527–0.673)	0.004	14.5	0.235	33.3	90.2
Predicted probability	0.873 (0.832–0.914)	0.000	0.2	0.586	84.4	74.2

AUC, area under the curve; PLT, platelet; LZD, linezolid.

### Validation of the Model and LZD Pharmacokinetic Parameters

We collected clinical data of 56 neonatal sepsis patients who received LZD therapy. Seventeen patients were subsequently excluded: seven patients received LZD for less than 5d and ten had no steady-state trough concentrations. Finally, 39 cases were included. According to the definition of LIT, nine neonates had LIT (*n* = 9, 23.7%). The general clinical data of the two groups are shown in Table 2. Compared with the non-LIT group, the LIT patients had lower GA and lower body weight at birth and medication, which was statistically significant (*p* = 0.019, *p* = 0.005, *p* = 0.008), similar to Part one of this study. The accuracy rates of positive predictive and negative prediction of LIT by this logistic regression model were 88.9 and 76.7% (Table 3).

**TABLE 2:** General clinical data of patients.

Characteristics	Value	LIT group (*n* = 9)	Non-LIT group (*n* = 30)	T/Z	*p* value
Gender	—	—	—	—	—
Female, n (%)	14 (35.9)	4 (44.4)	10 (33.3)	—	0.696
Male, n (%)	25 (64.1)	5 (55.6)	20 (66.7)		
GA (w), (mean ± SD)	31.3 ± 3.7	28.8 ± 3.3	32.0 ± 3.5	−2.463	0.019
PNA (d), median (IQR)	12.0 (7.0, 20.0)	10.0 (3.0, 26.5)	12.5 (8.8, 19.3)	−0.818	0.413
BW (g), median (IQR)	1350.0 (1000.0, 1850.0)	880.0 (760.0, 1360.0)	1525.0 (1200.0, 1900.0)	−2.836	0.005
Weight at medication (g), median (IQR)	1560.0 (1190.0, 1990.0)	840.0 (745.0, 1785.0)	1630.0 (1295.0, 2075.0)	−2.584	0.008
Duration of LZD treatments (d), median (IQR)	10.0 (9.0,12.0)	12.0 (8.0, 14.0)	10.0 (8.8, 11.0)	−1.197	0.231
PLT (×10^9^/L), median (IQR)	206.0 (169.0, 267.5)	236.0 (139.0, 312.0)	201.5 (169.0, 260.8)	−0.705	0.481

LZD, linezolid; LIT, LZD-induced thrombocytopenia; Non-LIT, non-LZD-induced thrombocytopenia; GA, gestational age; PNA, postnatal age; BW, birth weight; PLT, platelet.

**TABLE 3:** Dosage, trough concentration, and pharmacokinetic parameters of LZD in neonatal patients.

Parameters	LIT group	Non-LIT group	Z value	*p* value
Prediction correct rate of model	88.9%	76.7%	—	1.000
Trough concentration (mg/L), median (IQR)	11.49 (6.86, 15.13)	5.51 (2.80, 11.61)	−2.200	0.028
CL (L/h), median (IQR)	0.067 (0.023, 0.184)	0.114 (0.081, 0.149)	−1.100	0.271
Vd (L), median (IQR)	0.778 (0.687, 1.421)	1.322 (1.099, 1.610)	−2.584	0.010
AUC_24h_ (mg·h/L), median (IQR)	444.461 (163.378, 1103.221)	262.823 (201.195, 368.849)	−1.033	0.301

LZD, linezolid; LIT, LZD-induced thrombocytopenia; Non-LIT, non-LZD-induced thrombocytopenia; CL, clearance; Vd, volume of distribution; AUC_24h_, area under the curve.

**FIGURE 6:**
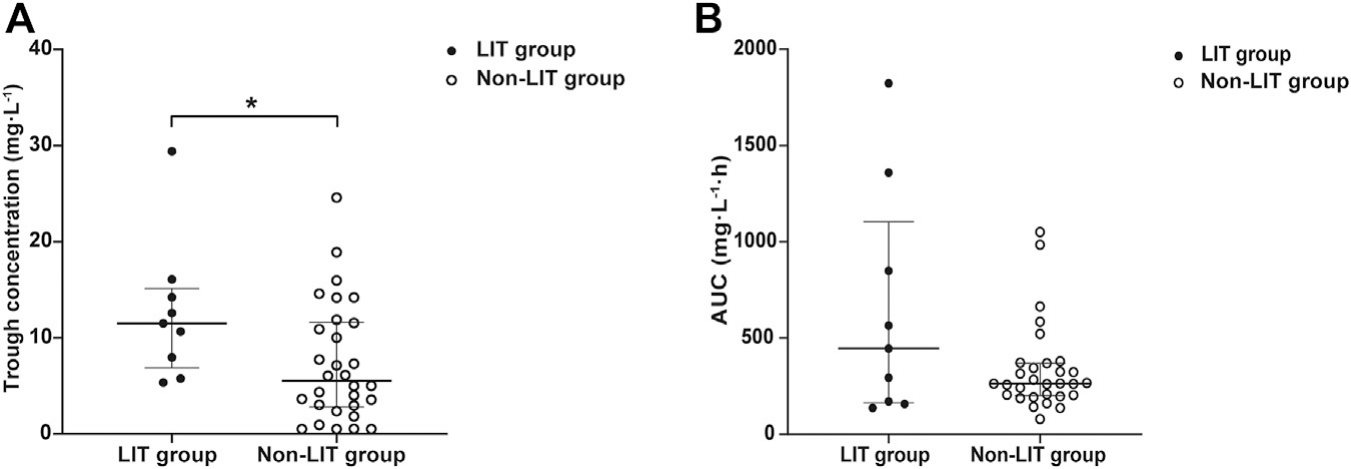
**(A)** Distribution of LZD initial trough concentration in the LIT group and non-LIT group. Compared with the non-LIT group, trough concentration was significantly higher in the LIT group [11.49 (6.86, 15.13) *vs*. 5.51 (2.80, 11.61), *p = 0.027]. **(B)** Distribution of LZD AUC_24h_ in the LIT group and non-LIT group.

The trough concentration and pharmacokinetic parameters of LZD are presented in Table 5. The initial trough concentration in the LIT group was significantly higher than that of the non-LIT group [11.49 (6.86, 15.13) *vs.* 5.51 (2.80, 11.61) mg/L; *p* = 0.028] (Figure 6). The Vd was significantly lower in the LIT group than that of the non-LIT group [0.778 (0.687, 1.421) *vs.* 1.322 (1.099, 1.610); *p* = 0.010]. The CL in the LIT group was lower, while the AUC_24h_ was higher, but there was no significant difference when compared with the non-LIT group (*p* = 0.271, *p* = 0.301) (Figure 6).

## Discussion

LIT is a common and serious adverse drug reaction in adults treated with LZD, especially higher in critically ill patients (Tang et al., 2016a; Crass et al., 2019; Giunio-Zorkin and Brown, 2019; Rao et al., 2020). Although dose adjustment based on weight was performed for pediatric patients, thrombocytopenia was also detected in 21.4% of pediatric patients (Ogami et al., 2019). In our study, the incidence of LIT in neonatal sepsis patients was 19.9%, similar to the reports of pediatric patients (Ogami et al., 2019). More than 80% of LIT patients were preterm infants, and some cases also had decreases in hemoglobin, white blood cell counts, or hyperlactic acidemia. This side effect could be a result of the immaturity of mitochondrial protein synthesis in preterm patients (Honzik et al., 2008; Thibault et al., 2019). This side effect is especially pertinent for preterm infants because they have some risk factors for hepatic dysfunction and renal dysfunction (growth retardation, immature renal function, patent ductus arteriosus, hemodynamic failure, and other toxic drug treatments) (Thibault et al., 2019). The overall median time from the initiation of therapy to the development of thrombocytopenia was much shorter in preterm infants when compared with term infants [10 (6, 12) *vs*. 13 (9.75, 16.5), *p* = 0.004], which might be related to the physiological changes of preterm infants.

In our study, the risk factors of LIT in neonatal patients were significantly different from critically ill adult patients (Chen et al., 2012; Giunio-Zorkin and Brown, 2019; Kim et al., 2019; Ogami et al., 2019). Based on our previous research, neonates with younger gestational ages, lower body weight, and hyperbilirubinemia were more likely to have LIT (Tang et al., 2016b). Therefore, it is necessary to establish a regression model to predict LIT in neonatal patients.

Most studies have reported that thrombocytopenia is associated with a high trough concentration of LZD, long course of LZD treatment, combined use of meropenem, low albumin level, low baseline of PLT, and renal insufficiency in the adult population (Chen et al., 2012; Giunio-Zorkin and Brown, 2019; Kim et al., 2019; Ogami et al., 2019). In our neonatal patients, albumin level and baseline of PLT were negatively correlated with the occurrence of LIT, days of LZD treatment, and having a positive correlation with the occurrence of LIT. However, no correlation was found between renal function and LIT. The protein binding rate of LZD was 31%, and hypoproteinemia could increase the free drug concentration. Adult patients receiving human serum albumin rarely developed LIT during LZD treatment. This result indicated that serum albumin might be a protective factor of LIT (Chen et al., 2012). Patients with a lower baseline of PLT and more days of LZD treatment were more likely to develop thrombocytopenia (Gerson et al., 2002; Chen et al., 2012). Chen et al. used the ROC curve to predict the cut-off point for LIT with good sensitivity and specificity. Results indicated that baseline platelet count ≤ 181 × 10^9^/L and duration of LZD treatment ≥ 10 days were independent risk factors for LIT (Chen et al., 2012). In our neonatal population, the cut-off values of the ROC curve were baseline platelet count ≤ 233 × 10^9^/L and duration of LZD treatment ≥ 14.5 days, which were different from the adult reports. Maybe it is due to the different diagnosis value of thrombocytopenia, 150 × 10^9^/L in neonates, which is physiologically different from that of adults. (Gunnink et al., 2014). The recommended duration of LZD treatment is 10–14 days. LZD can inhibit its metabolism, the clearance of LZD decreases with the prolonged treatment days, and prolonged use of LZD (>14 days) can increase the risk of thrombocytopenia (Gerson et al., 2002; Plock et al., 2007), which is consistent with our study.

Preterm patients have lower body weight and younger gestational ages, have inadequate drug metabolism and excretion due to the immature liver and renal function, and are more likely to develop drug accumulation (Honzik et al., 2008; Thibault et al., 2019). Thibault et al. (2019) established a PK model of LZD in preterm infants, indicating that PNA and weight positively correlated with LZD clearance. Preterm patients had lower drug clearance, lower body weight, and postnatal age and were more likely to have drug accumulation and adverse drug reactions (Honzik et al., 2008; Thibault et al., 2019), which could account for the correlation between preterm and LIT. LOS is an important risk factor for thrombocytopenia in the NICU (Ahmed et al., 2017). According to the onset time of thrombocytopenia, it is diagnosed as early-onset (within 72 h after birth) and late-onset thrombocytopenia (LOT). The common causes of neonatal LOT were NEC, bacterial or fungal sepsis, thrombosis, and medication (Gunnink et al., 2014). In our study, LOS, sepsis shock, purulent meningitis, and NEC were the risk factors of LIT, and LOS and NEC were the independent factors, similar to the other reports (Gunnink et al., 2014). The relationship between scores of Apgar at 1 min, respiratory failure, and mechanical ventilation with LIT has not been reported, and maybe these factors were related to the severity of the critically ill.

According to the risk factor analysis, many studies have established LIT detection methods, such as the prediction model of platelet changes in adult patients (Tsuji et al., 2017). In our study, the ROC curves for a single risk factor only had a moderate predictive value, so we established a regression model with multiple factors to identify the LIT in critically ill neonates. The predicted probability of this model had a ROC AUC of 0.873 (*p* < 0.001), and sensitivity and specificity were 84.4 and 74.2%, which indicated that the model had a high predictive value. Verification of 39 other critically ill neonatal patients who received LZD treatment showed that the predictive accuracy of this model for LIT prediction exceeded 75%, and it had a high predictive performance.

The initial trough concentration was significantly higher in the LIT group than that of the non-LIT group (*p* = 0.028), indicating that LIT has a certain correlation with the initial trough concentration of LZD. This result suggested that controlling the trough concentration within the target range could effectively reduce the risk of LIT. The Vd of the LIT group was significantly lower than that of the non-LIT group (*p* = 0.010). The published PPK model of LZD in preterm infants was a one-compartment model. A decrease in Vd may lead to an increase in the blood concentration of LZD on the basis of the same administration dose (Thibault et al., 2019). The Vd is positively correlated with body weight at medication according to the calculation formula, so the decreased Vd in the LIT group may be related to the lower body weight in the LIT group than that of the non-LIT group (*p* = 0.008). PNA and body weight were the main determinants of LZD CL in preterm infants although body weight at medication was significantly different between the two groups, but there was no difference in PNA. CL was lower and AUC was higher in the LIT group, but there was no significant difference when compared with the non-LIT group (*p* = 0.271, *p* = 0.301). A larger sample size is needed to evaluate the differences in pharmacokinetic parameters and their relationship with LIT.

However, there are a few limitations in this study: 1) our study analyzed risk factors of LIT in critically ill neonatal patients, but it is possible that not all impact factors are included. 2) This is a 10-year retrospective cohort study, and the entire data came from the neonatal medical database, which is representative of the Suzhou area, but there may still be biases. 3) In this retrospective study, the trough concentrations of LZD were not determined and could not be included as a variable in the LIT prediction regression model, which may lead to an inaccurate prediction. 4) This is a single-center clinical study, so the sample of critically ill neonatal patients treated with LZD is too limited to precisely estimate the pharmacokinetics of LZD. In the future, multicenter clinical cohort studies including more impact factors can be undertaken to analyze and evaluate risk factors of LIT in critically ill neonatal patients and the influence of the LZD trough concentration on LIT and clinical efficacy.

## Conclusion

In conclusion, the incidence of LIT was high in preterm infants. LIT occurred earlier in preterm infants than in term infants. The seven independent factors for the occurrence of LIT were lower weight at LZD medication, younger gestational ages, LOS, NEC, mechanical ventilation, lower baseline of PLT, and longer durations of LZD treatments. The regression model of seven variables had a high predictive value for predicting LIT. LIT was correlated with higher trough concentration and lower Vd, so controlling the trough concentration within the target range can effectively reduce the risk of LIT.

## Data Availability

The original contributions presented in the study are included in the article/Supplementary Material. Further inquiries can be directed to the corresponding authors.
